# The impact of COVID-19 lockdowns on physical activity amongst older adults: evidence from longitudinal data in the UK

**DOI:** 10.1186/s12889-022-14156-y

**Published:** 2022-09-22

**Authors:** Jack Elliott, Luke Munford, Saima Ahmed, Alison Littlewood, Chris Todd

**Affiliations:** 1 National Institute for Health and Care Research Applied Research Collaboration Greater Manchester, Manchester, UK; 2 Manchester Academic Health Science Centre, Manchester, UK; 3 Health Organisation, Policy and Economics, Division of Population Health, Health Services Research and Primary Care, School of Health Sciences, Faculty of Biology, Medicine and Health, The University of Manchester, Manchester, UK; 4 Division of Nursing, Midwifery and Social Work, School of Health Sciences, Faculty of Biology, Medicine and Health, The University of Manchester, Manchester, UK; 5 Manchester University NHS Foundational Trust, Manchester, UK; 6 Manchester Institute for Collaborative Research On Ageing, Manchester, UK

**Keywords:** COVID-19, Physical activity, Older adults, Health inequalities, Public health

## Abstract

**Background:**

A sedentary lifestyle increases the risk of adverse health outcomes and frailty,particularly for older adults. To reduce transmission during the COVID-19 pandemic, people were instructed to stay at home, group sports were suspended, and gyms were closed, thereby limiting opportunities for physical activity. Whilst evidence suggests that physical activity levels reduced during the pandemic, it is unclear whether the proportion of older adults realising the recommended minimum level of physical activity changed throughout the various stages of lockdown.

**Methods:**

We used a large sample of 3,660 older adults (aged ≥ 65) who took part in the UK Household Longitudinal Study’s annual and COVID-19 studies. We examined changes in the proportion of older adults who were realising the UK Chief Medical Officers’ physical activity recommendations for health maintenance at several time points before and after COVID-19 lockdowns were imposed. We stratified these trends by the presence of health conditions, age, neighbourhood deprivation, and pre-pandemic activity levels.

**Results:**

There was a marked decline in older adults’ physical activity levels during the third national lockdown in January 2021. The proportion realising the Chief Medical Officers’ physical activity recommendations decreased from 43% in September 2020 to 33% in January 2021. This decrease in physical activity occurred regardless of health condition, age, neighbourhood deprivation, or pre-pandemic activity levels. Those doing the least activity pre-lockdown increased their activity during lockdowns and those doing the most decreased their activity levels.

**Conclusions:**

Reductions in older adults’ physical activity levels during COVID-19 lockdowns have put them at risk of becoming deconditioned and developing adverse health outcomes. Resources should be allocated to promote the uptake of physical activity in older adults to reverse the effects of deconditioning.

**Supplementary Information:**

The online version contains supplementary material available at 10.1186/s12889-022-14156-y.

## Introduction

Physical activity refers to any bodily movement made by the skeletal muscles that requires energy expenditure and it includes, for example, movements done during leisure time, for transport to get to and from places, or as part of a person’s work [1].  Regular physical activity that raises your heart rate and makes you breathe faster is consistently associated with reduced risk of chronic diseases [2],  cognitive decline [3],  and mortality [4]. This is particularly true of older populations, amongst whom time spent not physically exerting is associated with frailty and adverse health outcomes [5], and sitting for 8 h or more increases the risk of all-cause mortality [6]. The UK Chief Medical Officers (CMOs) recommend that older adults do activities to maintain or develop muscle strength at least twice a week and accumulate 150 min of moderate-intensity aerobic activity (i.e., activities that leave you unable to sing afterward, such as brisk walking or cycling) or 75 min of vigorous-intensity activity (i.e., activities that make it difficult to talk afterward without pausing, such as running), or a combination of two. They should also avoid sedentary behaviours (e.g., watching television, reading, working with a computer) and break up prolonged periods of inactivity with light activity (e.g., cleaning, carrying out rubbish, yoga) [7].

During the COVID-19 pandemic, the UK government instigated several public health measures to reduce transmission. Key amongst these measures were a series of national lockdowns and regional restrictions, whereby people were mandated to remain at home except for specific essential reasons [8–10]. Further detail on the restrictions imposed by each of the four nations in the UK, and compliance with these restrictions, is provided in ‘Additional File 1’.

Whilst recognising the need for such measures to reduce transmission and prevent COVID-19 deaths, there were concerns that measures could have adverse effects on the physical and mental health of the population, especially amongst the most vulnerable; older adults and those with single or multiple morbidities. These adverse health effects could have been worsened by the temporary suspension of group sports, fitness classes, and the use of gyms, limiting opportunities to engage in physical activity. Even when it was permitted, older adults could have consciously decided to refrain from group physical activity to reduce the risk of exposing themselves to COVID-19, a disease for which they are particularly vulnerable [11].

Existing evidence from the international literature suggests that people from a range of populations became less active and/or increased sedentary activities during the pandemic [12–18]. Considering older adults, evidence from a German and an international study both suggest that containment measures from the early stages of the pandemic (April-June 2020) had a deleterious effect on energy expenditure and frequency of physical activity [14, 18].

In England, evidence from the first national lockdown suggests that population activity declined and that this was particularly pronounced for older adults [19, 20]. For example, Public Health England documented that the rate of physical inactivity among older adults had increased from 27% in March to May 2019 to 32% in March to May 2020 and modelled that this would lead to an increase in deconditioning as well as 110,000 extra people having at least one fall per year [19].

In this study, we aim to determine whether older adults were put at risk of adverse health events because of lockdown-related activity reduction and whether these risks were more prevalent for certain subgroups. We did so by examining the proportion of older adults realising the Chief Medical Officers’ physical activity recommendations at several time points before and during COVID-19 lockdowns using a large nationally representative UK dataset, stratifying the population by health status, age, and level of deprivation.

## Methods

### Data: UK Household Longitudinal Study

We used data from UK Household Longitudinal Study (UKHLS) [21–24]. *UKHLS* is a nationally representative, longitudinal study of people in the UK that started in 2009 and comprises approximately 40,000 households. *UKHLS* contains a rich set of information relating to many aspects of respondents’ lives. The main survey is conducted annually with a two-year rolling window making up one wave.

From April 2020, selected participants were asked to complete monthly (every two months from September 2020) web-based surveys examining the impact of the COVID-19 pandemic on the welfare of UK individuals, families, and wider communities.

This paper utilises study waves that contain data on the participants’ physical activity. These include waves 7 (Jan 2015-Jun 2017), 9 (Jan 2017-Jun 2019), and 11 (Jan 2019-December 2021) from the annual *UKHLS* main survey and waves 1 (April 2020), 5 (September 2020), and 7 (January 2021) from the COVID-19 study. We dropped all observations from the annual wave 11 data that were collected after February 2020 to avoid capturing any effects of COVID-19.

### Measures

#### Physical activity

Participants were asked to provide information on the number of days they were physically active for at least ten minutes at a time in the last seven days for three different types of physical activity: walking, moderate physical activity, and vigorous physical activity. Walking included that done “at work and at home, walking to travel from place to place, and any other walking that you might do solely for recreation, sport, exercise, or leisure”; moderate activities include those that “make you breathe somewhat harder than normal and may include carrying light loads, bicycling at a regular pace, or doubles tennis”; and vigorous activities include those that “make you breathe much harder than normal and may include heavy lifting, digging, aerobics or fast bicycling.”

Respondents who indicated they were physically active for ten minutes or more on at least one day were also asked how much time they spent being physically active (for each activity type) in a combination of hours and minutes on one of those days. Full details of survey methods are published by *UKHLS* [25].

We defined two binary variables to proxy whether individuals realised the physical activity levels recommended by the CMOs (henceforth referred to as being ‘physically active’). The first of which was the primary focus of our analysis and entailed coding individuals as 1 (Yes) if an individual completed at least 150 min of moderate physical activity *or* at least 75 min of vigorous physical activity per week. However, unlike *UKHLS*, the CMOs’ definition of moderate physical activity includes “brisk walking.” Hence, we also derived an alternative proxy variable where individuals were coded as 1 (Yes) if they completed at least 150 min of walking or moderate physical activity, or at least 75 min of vigorous physical activity. Individuals with missing data for total minutes of vigorous or moderate activity (or walking for alternative proxy variable) were not included in the analysis.

We derived an estimate of participants’ total minutes of physical activity per week for each type of activity by multiplying the reported total minutes of each activity by the number of days reportedly active. Full details of the questions and response options used to determine the participants’ physical activity in *UKHLS* annual and COVID-19 questionnaires are provided in ‘Additional File 2’.

We were able to determine whether 15,568 individuals (14,523 for alternative proxy variable) were physically active in at least one of the annual waves of data *and* at least one of the waves in the COVID-19 study. Of these, 3,660 (3,312 for alternative proxy variable) reported being aged 65 or over before March 2020. This subset of individuals was used as the core sample. Sociodemographic descriptive statistics of this sample are reported in ‘Additional File 3’.

#### Additional variables

*UKHLS* contains data relating to individual characteristics that could feasibly influence how lockdown affects trends in physical activity, and thus lead to widening pre-existing health inequalities. To examine differences in physical activity trends, we used data on respondent age and whether they had a self-reported long-standing physical or mental impairment, illness, or disability in at least one of the pre-COVID-19 annual waves of data. We also examined differences by stratifying the sample into tertiles based on individuals’ average level of physical activity across the pre-COVID-19 waves of data.

To examine differences in physical activity trends by neighbourhood deprivation, we used Office of National Statistics data containing information on respondents’ index of multiple deprivation (IMD; a scale from most deprived to least deprived) deciles for England (2019), Wales (2019), Scotland (2020) and Northern Ireland (2017). We used Lower Layer Super Output Area (LSOA) codes, or national equivalents, to link these to the UKHLS data.

We defined a ‘least deprived’ population and a ‘most deprived’ population. An individual was defined as belonging to the most deprived population if, for the respective nations (England, Scotland, Wales, Northern Ireland), the LSOA or equivalent they reside in has IMD ranking (from April 2020) below the median.

There were differing levels of missing data across the questions used to derive these additional variables. Therefore, where we stratified the sample, the sample size varies slightly across different analyses. ‘Additional File 4’ illustrates how the samples of respondents were determined.

### Analysis

#### Empirical approach

We examined trends in the proportion of older individuals who were physically active both before and after the government-imposed lockdown in March 2020.

#### Stratifying by respondent characteristics

To examine how the trends varied by individual characteristics, and how lockdowns could have widened existing inequalities, we re-examined the trends after stratifying the sample by whether respondents (i) had a health condition, (ii) respondent age, (iii) the level of deprivation. Where the sample was stratified by age, we also examined trends for a comparator group of individuals who were aged 16–64 before March 2020.

Any change in the proportion of individuals who were physically active after the COVID-19 lockdown is an aggregation of those who were already physically active before lockdown and those that were more sedentary. The effect of lockdown is not necessarily homogenous for these groups and taking a simple average could mask these important differences. Hence, to further investigate differences among certain populations, we re-examined the trends after stratifying the sample into tertiles based on respondents’ total minutes of moderate activity and vigorous activity per week (i.e., the measures used to dichotomise the variable on whether physically active), averaged across pre-COVID-19 waves.

#### Seasonality

The season can affect activity levels, generally with lower levels of activity in the winter months and higher in summer [26]. This is largely driven by the climate and day length where, for example, the UK’s colder temperatures, greater levels of rainfall, and fewer sunshine hours in the winter months [27] are associated with less physical activity among older adults [28].

Given that before the introduction of the lockdown, data were collected over the year and after lockdown data were collected for specific months, changes in activity levels could be reflecting seasonality rather than the effect of an imposed lockdown. Hence, we re-examined the trends described, restricting the sample to respondents from whom data were collected one month on either side of April, September, and January.

## Results

### Main analysis

**Fig. 1:**
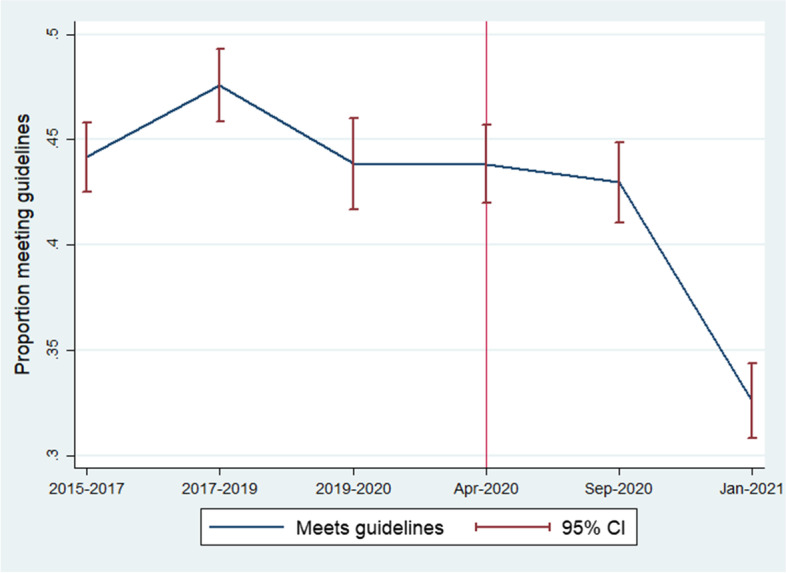
Proportion of older adults (aged 65 years and older) meeting physical activity guidelines over time (red vertical line indicates the introduction of the first UK lockdown)

Figure 1 describes the trend in the proportion of individuals aged 65 years or older who were physically active before and during the COVID-19 lockdown, in terms of meeting CMO guideline levels of activity. The proportion of individuals achieving CMO guidelines on physical activity remains constant with overlapping 95% confidence intervals from before the COVID-19 lockdown up until September 2020. From September 2020 to January 2021, the month in which England’s third national lockdown was imposed, there was a noticeable decrease in the proportion of physically active individuals, such that confidence intervals no longer overlapped. Where the alternative proxy was used, a similar decrease in physically active individuals was observed from September 2020 to January 2021 (see ‘Additional File’ 5).

### Stratifying by respondent characteristics

**Fig. 2:**
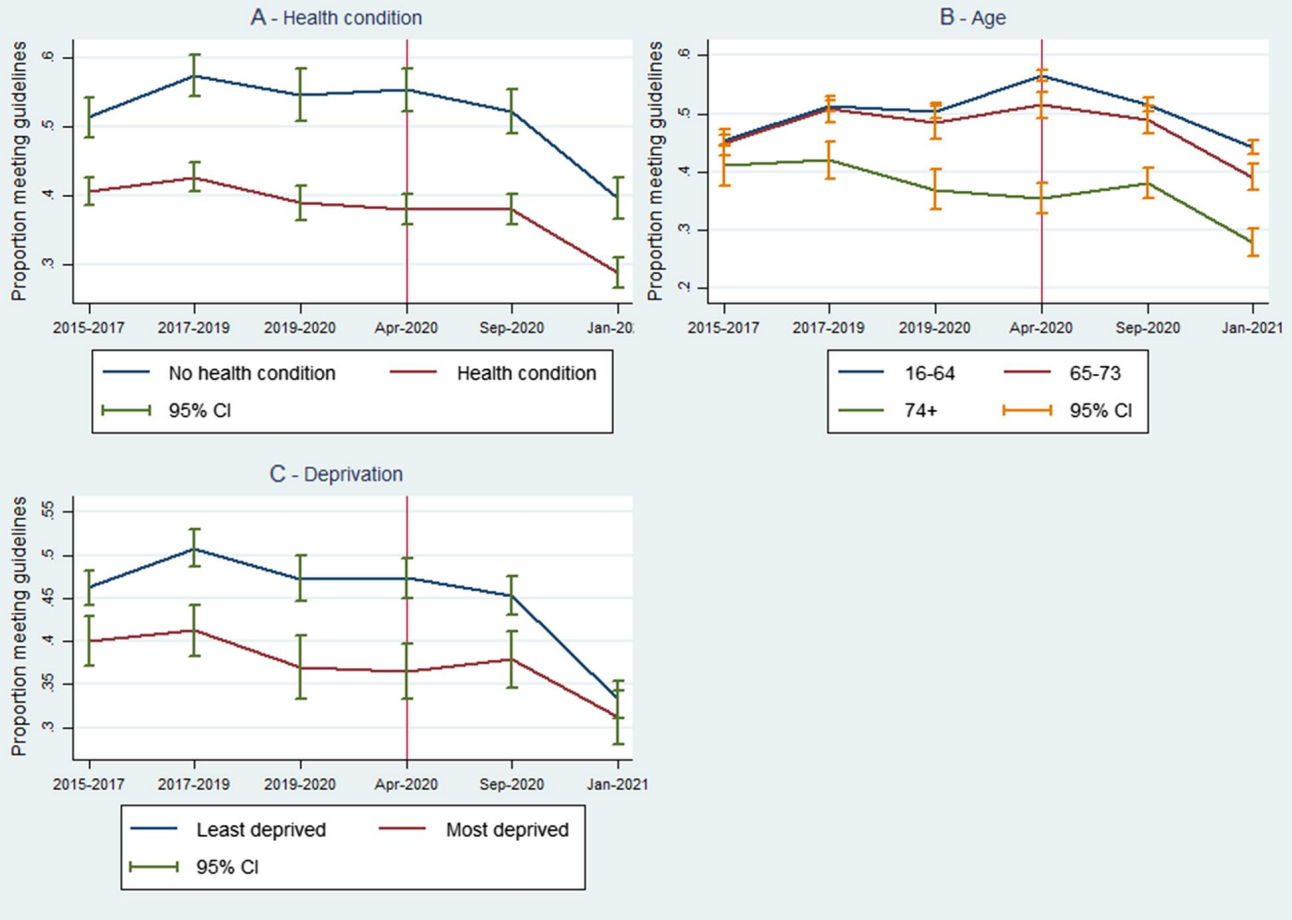
Proportion of older adults (aged 65 years and older) meeting physical activity guidelines over time, stratified by subgroups (Panel **B** includes data from 16–64 age group for comparison; red vertical line indicates the introduction of the first UK lockdown)

Figure 2 presents the proportion of physically active individuals stratifying the sample by health condition, age, and level of deprivation (in Panels A, B, and C respectively).

As expected, the proportion of those with a health condition who were physically active was lower than the proportion of those without a health condition. The trends were largely parallel for both populations aside from those without a health problem experiencing a larger decrease in the proportion from September 2020 to January 2021.

The oldest stratum of the population (74 +) had the smallest proportion of physically active individuals. Immediately after the first national lockdown was imposed (April 2020), the youngest part of the population (16–64) showed a temporary increase in the proportion engaged with physical activity. From September 2020 to January 2021, the oldest sub-group also demonstrated a larger decrease in physical activity levels despite them already having a low proportion relative to the other age groups.

The proportion of physically active individuals was smaller amongst those living in more deprived areas. The trends across the least deprived and most deprived parts of the population were close to parallel. From September 2020 to January 2021, however, there was a more substantial decrease in the proportion of physically active individuals for the least deprived parts of the population.

**Fig. 3:**
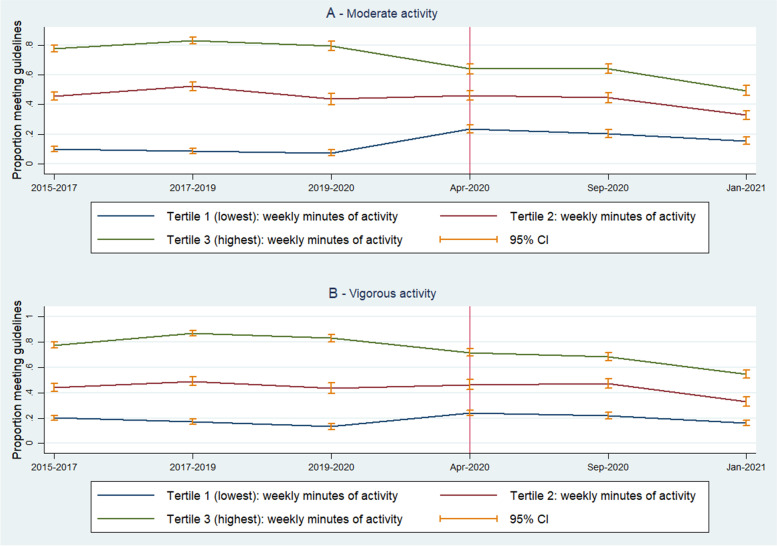
Proportion of older adults (aged 65 years and over) meeting physical activity guidelines over time, stratified by baseline physical activity behaviours in tertiles (red vertical line indicates the introduction of the first UK lockdown)

Figure 3 presents the proportion of physically active individuals stratified by respondents’ pre-lockdown weekly minutes of moderate and vigorous activity (see Panel A and B, respectively). For both levels of physical activity, it seems that those who were least active before the pandemic (Tertile 1) were more likely to increase their activity levels after COVID-19 restrictions were introduced. Conversely, those who spent on average the most weekly minutes engaged in physical activity pre-pandemic (Tertile 3) were more likely to experience a decrease in physical activity.

### Seasonality

After restricting the sample to data collected in April, September, and January (and allowing for one month on either side), the proportion of physically active individuals generally decreased after COVID-19 restrictions were imposed, although with overlapping confidence intervals. This decrease was observed for the physical activity measures that exclude and include walking (see ‘Additional File 6’ and ‘Additional File 7’, respectively).

## Discussion

### Main findings

To examine whether COVID-19 and the associated lockdowns have put older adults at risk of adverse health events caused by reduced activity levels, we observed the proportion of individuals who were realising CMOs’ weekly physical activity recommendations both before and after COVID-19 lockdowns were introduced. We defined an individual as realising the CMO guidelines if they completed at least 150 min of moderate physical activity *or* at least 75 min of vigorous physical activity per week.

To examine whether any subgroups were particularly at risk of reduced physical activity, we also stratified our analysis by self-reported health condition, age, deprivation, and baseline physical activity level.

Our core findings are that for adults aged 65 and over:

- Activity levels remained about the same as pre-pandemic at the beginning of the pandemic despite the imposition of the first lockdown, but there was a marked decline in activity between September 2020 and January 2021, with a drop from 43 to 33% of people achieving the CMOs’ recommended levels of physical activity.
- Those least active before the beginning of the COVID-19 pandemic became more active whilst the most active became less active, with lower activity in April 2020 during the first national lockdown (see Fig. 3) with continued decline thereafter.
- Whilst decreases in activity levels are seen across the social spectrum, the reduction in activity level between September 2020 and January 2021 is most marked amongst those from the least deprived areas. Those from the most deprived areas also decreased their activity levels and remain the least active group.
- Likewise, whilst those reporting no health condition had more marked decreases in activity levels to January 2021 than those with health conditions, those with health conditions also have considerably lower levels of activity during the pandemic than pre-pandemic and these are lower than people with no health conditions.
- Individuals aged 74 years and over before March 2020 had more marked decreases in activity levels to January 2021 than those aged 65–73, despite the older cohort having lower levels of activity pre-pandemic.

At the beginning of the pandemic, there was considerable public health messaging about the need to stay active despite the regulations around having only one hour of outdoor public activity per day. During the early days of the pandemic, the weather was unusually good for the time of year across the UK, and many were determined to get their allotted hour of outdoor exercise [29]. The least active becoming more active could perhaps be explained by this allotted hour being more than they usually got, but for the most active an hour was a reduction of their normal level (79% of the most active tertile achieved CMO guidelines on moderate activity pre-lockdown but by January 2021 only 49% did so, a large change in activity patterns). It is also plausible that the least active were more motivated to increase activity to mitigate the risks of complications from COVID-19 infection because maintaining their lower baseline levels would have made them susceptible [30]. As the months went on the commitment to a regimen may have changed, as did the weather, reflected perhaps in overall reductions in activity. No doubt continued fears about COVID-19 transmission and the psychological effects of home isolation took their toll on people’s motivation to remain active.

By January 2021, activity levels reduced for all regardless of age, pre-pandemic activity level, health condition, or level of deprivation. Although the levels of change may have been greatest for the least deprived, healthiest and most active parts of society, the absolute effect can be seen as most severe amongst the oldest, least healthy, least active, and most deprived sectors of society, amongst whom only 16–31% were achieving CMOs’ activity guidelines by January 2021.

In deciding whether to engage in physical activity amid the COVID-19 pandemic, older adults must weigh up the benefits and risks to their health. Whilst regular activity can reduce the risk of chronic disease, [2] mortality, [4] and deconditioning, [19] and help mitigate against complications from COVID-19; [31] activity conducted with others, especially in an indoor setting, increases the probability of exposure [32]. These trade-off determinations are everchanging and will depend on the infection rate and the presence of new variants. For example, the Omicron variant of the virus, which arrived in the UK after our study period, in November 2021, [33] is more transmissible but causes less severe disease than prior variants [34]. This will likely have affected older adults’ decision to be physically active.

Going forward, older adults must be encouraged to engage in regular physical activity in order to reverse the negative health effects caused by lockdown-related inactivity. To do this effectively, it is integral that measures are taken to minimise the risk of COVID-19 infection, and that activity remains accessible to those who wish to socially distance themselves, especially in the winter months when the climate makes outdoor activity less feasible. Firstly, to reduce the risk of transmission in premises hosting indoor physical activity, we recommend the use of air filtration devices, implementing regular airing breaks, and encouraging participants to test for COVID-19 before entry [32, 35]. Secondly, we recommend that government officials, health experts, and media professionals promote the uptake of home workouts so those wishing to avoid group physical activity can be active in the winter — tutorial videos can be accessed online and often do not require any equipment [36].

### Strengths and limitations

This study is based on a large longitudinal nationally representative dataset. The longitudinal nature of UKHLS, and its COVID-19 waves during 2020–2021, allowed us to analyse temporal changes in physical activity related to the COVID-19 pandemic. The collection of activity variables in UKHLS has allowed us to map these changes to CMOs’ physical activity recommendations for maintaining health. Dropping below those levels is very likely to result in deconditioning with long-lasting health effects [19]. Indeed, the data presented here provide further and independent evidence that the Public Health England estimates [19] are likely to be correct.

However, our study does have some limitations. Firstly, we cannot interpret changes in activity as being necessarily caused by lockdowns. As this is an observational study, we can only speculate on the mechanisms causing decreases in physical activity and the causes of differences between sub-groups.

Secondly, while we took measures to control for seasonality in our analysis, the confidence intervals for the estimates that restricted analysis to data collected in a particular month are large, and we were unable to control for the weather. More rainfall in a particular month during Covid-19 lockdowns compared to the same month in a previous year could be explaining the decrease in the proportion of physically active individuals.

Finally, the questionnaires used in the *UKHLS* Covid-19 study were conducted via the web, whereas the questionnaires in the annual study were conducted face-to-face, via the web, or via the telephone. If there were systematic differences in activity levels between face-to-face and telephone respondents compared to the web, we could be misattributing changes in physical activity to COVID-19 and the associated lockdowns.

## Conclusions

There have been considerable changes in activity levels amongst older adults in the UK associated with the imposition of COVID-19 public health restrictions. Whilst these restrictions helped control the spread of COVID-19, they have also likely had adverse effects on population health including deconditioning from reduced activity levels. It is therefore important that resources are allocated to help older adults regain pre-pandemic activity levels to counteract the long-term health effects associated with deconditioning.

## Supplementary Information

**Additional file 1. **UK COVID-19 restrictions from March 2020 to January 2021 [37–53].

**Additional file 2.** Questions and response options used to derive health behaviours.

**Additional file 3.** Sociodemographic descriptive statistics of core sample.

**Additional file 4. **Data flow and sample derivation.

**Additional file 5. **Proportion of older people (age 65 years and older) meeting physical activity guidelines over time using the alternative proxy for physical activity.

**Additional file 6. **Proportion of older people (age 65 years and older) meeting physical activity guidelines over time, restricted to the month of data collection.

**Additional file 7. **Proportion of older people (age 65 years and older) meeting physical activity guidelines over time using the alternative proxy for physical activity, restricted to the month of data collection.

## Data Availability

The data underlying this article are available in UK Data Service, at http://doi.org/10.5255/UKDA-SN-6614-16 and http://doi.org/10.5255/UKDA-SN-8644-11. The data used to link these to geographical identifiers are available in UK Data Service, at http://doi.org/10.5255/UKDA-SN-7248-11 and http://doi.org/10.5255/UKDA-SN-8663-7. Access to these data require a special license from the UK Data Service. The Stata code used to clean and analyse the data used in this article will be shared on reasonable request to the corresponding author.

## Abbreviation

CMO: Chief Medical Officer
