# Supplementary material for: The impact of COVID-19 lockdowns on physical activity amongst older adults: evidence from longitudinal data in the UK
Source: BMC Public Health. 2022 Sep 22;22:1802. doi: 10.1186/s12889-022-14156-y (PMC9502942; doi:10.1186/s12889-022-14156-y)
Supplement: Supplementary file 1 — Additional file 1. UK COVID-19 restrictions from March 2020 to January 2021 [37–53]. [file 12889_2022_14156_MOESM1_ESM.docx]

**Additional File 1**

UK COVID-19 restrictions from March 2020 to January 2021

On 23 March 2020, the UK Government and devolved administrations went into lockdown,(37) and stay-at-home orders were imposed restricting all but essential travel, shutting down most education establishments and businesses, and restricting gatherings.(8) People were required to stay at home except when essential — those belonging to exempt specific occupation groups (i.e., health and social care and other critical occupations), for medical requirements, for care provision, or shopping for basic necessities. Exercising outside the home was initially only allowed for one hour per day, alone or with your household.(37)

Across the four nations, restrictions on outdoor recreation were relaxed in May 2020 but essentially required maintenance of social distancing, and gatherings were still banned. By June 2020 restrictions on outdoor gatherings had started to ease provided social distancing was maintained: gatherings of six people were permitted in England and Northern Ireland, eight people across two households in Scotland, and two households in Wales.(10,38–40) From June to September 2020, there was further easing of restrictions across all four nations that included the re-opening of pubs, restaurants, non-essential retail outlets, schools, and gyms.(39,41,42) In September 2020, restrictions on gatherings were tightened and local restrictions imposed. In October 2020, national tier systems were introduced in England and Scotland, with more severe restrictions imposed on areas with higher COVID-19 rates in an attempt to curb transmission.(43,44) In the same month, Wales and Northern Ireland introduced multi-week ‘circuit breaker’ lockdowns to slow the spread of the virus, which required people to stay at home except for very limited purposes, such as visiting public parks.(45,46) In November 2020, England introduced a second month-long national lockdown restricting going out for non-essential reasons.(47) After some easing of restrictions around Christmas, all of the UK went into a third lockdown. This lockdown gradually started to be relaxed from March 2021, following announcements of lockdown exit strategies from the respective administrations.(48–50)

Compliance with the imposed restrictions was generally high, especially amongst older adults and in the early stages of the pandemic.(51–53) Younger people were less likely to comply with restrictions on social mixing, especially as temperatures increased and restrictions became less stringent.(51–53)
