# Supplementary material for: The impact of COVID-19 lockdowns on physical activity amongst older adults: evidence from longitudinal data in the UK
Source: BMC Public Health. 2022 Sep 22;22:1802. doi: 10.1186/s12889-022-14156-y (PMC9502942; doi:10.1186/s12889-022-14156-y)
Supplement: Supplementary file 2 — Additional file 2. Questions and response options used to derive health behaviours. [file 12889_2022_14156_MOESM2_ESM.docx]

**Additional File 2**

Questions and response options used to derive health behaviours

| Name | Text | Scripting notes | Universe |
| --- | --- | --- | --- |
| 7 days vigorous activity | Now, think about all the vigorous activities which take hard physical effort that you did in the last 7 days. Vigorous activities make you breathe much harder than normal and may include heavy lifting, digging, aerobics, or fast bicycling. Think only about those physical activities that you did for at least 10 minutes at a time. During the last 7 days, on how many days did you do vigorous physical activities?  During the last 7 days, on how many days did you do vigorous physical activities like heavy lifting, digging, aerobics, or fast bicycling? [Numeric] days per week | Visually separate the first paragraph from the question. Display a radio button on-screen with a label “No vigorous physical activities ^a)^”. Range [0 – 7]. | Ask All |
| Usual hours vigorous activities | How much time did you usually spend doing vigorous physical activities on one of those days? This is for physical activities done for at least 10 minutes at a time. [Numeric] hours per day | Display text at “Don't know” as “Don't know/Not sure”. Vdhrs and vdmin appear on the same screen. Range [0 – 16]. | If respondent performed vigorous activities in the last 7 days |
| Usual minutes vigorous activities | n/a [Numeric] minutes per day | Display text at “Don’t know” as “Don't know/Not sure”. Vdhrs and vdmin appear on the same screen. Range [0 – 59] | If respondent performed vigorous activities in the last 7 days |
| 7 days moderate activity | Think about all the moderate activities that you did in the last 7 days. Moderate activities refer to activities that take moderate physical effort and make you breathe somewhat harder than normal. Think only about those physical activities that you did for at least 10 minutes at a time.  During the last 7 days, on how many days did you do moderate physical activities like carrying light loads, bicycling at a regular pace, or doubles tennis? Do not include walking. [Numeric] days per week | Visually separate the first paragraph from the question. Display a radio button on-screen with a label “No moderate physical activities ^a)^”. Range [0 – 7]. | Ask All |
| Usual hours moderate activities | How much time did you usually spend doing moderate physical activities on one of those days? | Display text at “Don't know” as “Don't know/Not sure”. Mdhrs and mdmin appear on the same screen. Range [0 – 16]. | If respondent performed moderate activities in the last 7 days |
| Usual minutes moderate activities | n/a [Numeric] minutes per day | Display text at “Don't know” as “Don't know/Not sure”. Mdhrs and mdmin appear on the same screen. Range [0 – 59]. | If respondent performed moderate activities in the last 7 days |
| 7 days walking | Now think about the time you spent walking in the last 7 days. This includes at work and at home, walking to travel from place to place, and any other walking that you might do solely for recreation, sport, exercise, or leisure.  During the last 7 days, on how many days did you walk for at least 10 minutes at a time? [Numeric] days per week | Visually separate the first paragraph from the question. Display text at “Don't know” as “Don't know/Not sure”. Please display a radio button on-screen with a label “No walking ^a)^”. Range [0 – 7]. | Ask All |
| Usual hours walking | How much time did you usually spend walking on one of those days? Think only about the walking done for at least 10 minutes at a time. [Numeric] hours per day | Display text at “Don't know” as “Don't know/Not sure”. Wdhrs and wdmin appear on the same screen. Range [0 – 16]. | If walked in the last 7 days |
| Usual minutes walking | Text: n/a [Numeric] minutes per day | Display text at “Don't know” as “Don't know/Not sure”. Wdhrs and wdmin appear on the same screen. Range [0 – 59]. | If walked in the last 7 days |

Notes: ^a)^ For the annual questionnaire, those that clicked the on screen radio button were coded as value 0 and for the COVID-19 questionnaire, they were coded as -3. We re-coded those that clicked the on screen radio button during the COVID-19 questionnaire as value 0.
