# Supplementary material for: The impact of COVID-19 lockdowns on physical activity amongst older adults: evidence from longitudinal data in the UK
Source: BMC Public Health. 2022 Sep 22;22:1802. doi: 10.1186/s12889-022-14156-y (PMC9502942; doi:10.1186/s12889-022-14156-y)
Supplement: Supplementary file 3 — Additional file 3. Sociodemographic descriptive statistics of core sample. [file 12889_2022_14156_MOESM3_ESM.docx]

**Additional File 3**

Sociodemographic descriptive statistics of core sample

|  | 2015-2017 | 2017-2019 | 2019-2020 | April 2020 | September 2020 | January 2021 |
| --- | --- | --- | --- | --- | --- | --- |
| ***Characteristics*** |  |  |  |  |  |  |
| **Age [years, mean (Standard deviation)]** | 68.6 (5.4) | 70.5 (5.5) | 72.0 (5.6) | 72.9 (5.5) | 73.2 (5.2) | 73.4 (95.2) |
| **Age (range)** | 60-93 | 62-95 | 65-97 | 65-96 | 65-98 | 66-97 |
| **Long-standing illness or disability between 2015 and February 2020 [count, (%)]** | 2298 (65.6%) | 2095 (65.8%) | 1370 (67.8%) | 1822 (65.9%) | 1717 (64.6%) | 1709 (63.9%) |
| **IMD decile [count, (%)]** |  |  |  |  |  |  |
| 1 (Most deprived) | 109 (3.1%) | 103 (3.2%) | 69 (3.4%) | 93 (3.4%) | 79 (3.0%) | 77 (2.9%) |
| 2 | 187 (5.3%) | 179 (5.6%) | 104 (5.1%) | 144 (5.2%) | 136 (5.1%) | 129 (4.8%) |
| 3 | 205 (5.9%) | 173 (5.4%) | 112 (5.5%) | 161 (5.8%) | 136 (5.1%) | 146 (5.5%) |
| 4 | 262 (7.5%) | 249 (7.8%) | 150 (7.4%) | 201 (7.3%) | 209 (7.9%) | 205 (7.7%) |
| 5 | 370 (10.6%) | 353 (11.1%) | 226 (11.2%) | 280 (10.1%) | 274 (10.3%) | 286 (10.7%) |
| 6 | 395 (11.3%) | 343 (10.8%) | 206 (10.2%) | 309 (11.2%) | 289 (10.9%) | 294 (11.0%) |
| 7 | 448 (12.8%) | 393 (12.3%) | 242 (12.0%) | 340 (12.3%) | 347 (13.1%) | 341 (12.7%) |
| 8 | 467 (13.3%) | 431 (13.5%) | 271 (13.4%) | 385 (13.9%) | 359 (13.5%) | 352 (13.2%) |
| 9 | 510 (14.6%) | 449 (14.1%) | 293 (14.5%) | 394 (14.3%) | 388 (14.6%) | 407 (15.2%) |
| 10 (Least deprived) | 549 (15.7%) | 510 (16.0%) | 347 (17.2%) | 454 (16.4%) | 439 (16.5%) | 439 (16.4%) |
| **Male [count, (%)]** | 1725 (49.3%) | 1595 (50.1%) | 1002 (49.6%) | 1370 (49.6%) | 1322 (49.8%) | 1326 (49.6%) |
| **Lives alone between 2015 and February 2020 [count, (%)]** | 825 (23.6%) | 726 (22.8%) | 471 (23.3%) | 616 (22.3%) | 587 (22.1%) | 594 (22.2%) |
| ***Weekly physical activity*** |  |  |  |  |  |  |
| **Weekly minutes of moderate activity [mean, (Standard deviation)]** | 188.0 (385.1) | 217.2 (405.6) | 203.3 (393.4) | 218.0 (413.6) | 195.6 (369.6) | 139.4 (329.5) |
| **Weekly minutes of vigorous activity [mean, (Standard deviation)]** | 104.0 (298.2) | 145.3 (325.3) | 133.7 (324.6) | 181.7 (408.7) | 125.3 (287.8) | 62.7 (172.3) |
| **Observations**^†^ | 3502 | 3184 | 2020 | 2765 | 2656 | 2676 |

^†^ Includes those for whom data on realising physical activity recommendations were collected in the corresponding wave of data and in at least one of both the annual and COVID-19 waves of data.
