# Supplementary material for: The impact of COVID-19 lockdowns on physical activity amongst older adults: evidence from longitudinal data in the UK
Source: BMC Public Health. 2022 Sep 22;22:1802. doi: 10.1186/s12889-022-14156-y (PMC9502942; doi:10.1186/s12889-022-14156-y)
Supplement: Supplementary file 4 — Additional file 4. Data flow and sample derivation. [file 12889_2022_14156_MOESM4_ESM.docx]

**Additional File 4**

Data flow and sample derivation

Indicated total days of moderate activity
(N=14989 (2015-17), N=15695 (2017-2019), N=10331 (2019-20), N=15819 (April 2020), N=12154 (Sep 2020), N=11302 (Jan 2021))

Indicated both hours and minutes of moderate activity
(N=14336 (2015-17), N=14394 (2017-2019), N=8698 (2019-20), N=13910 (April 2020), N=10925 (Sep 2020), N=10938 (Jan 2021))

Indicated total days of vigorous activity
(N=15070 (2015-17), N=15805 (2017-2019), N=10426 (2019-20), N=15933 (April 2020), N=12228 (Sep 2020), N=11356 (Jan 2021))

Indicated both hours and minutes of vigorous activity
(N=14438 (2015-17), N=14429 (2017-2019), N=8981 (2019-20), N=13871 (April 2020), N=11073 (Sep 2020), N=10650 (Jan 2021))

Data on weekly minutes of moderate activity
(N= 14336 (2015-17), N=14394 (2017-2019), N=8698 (2019-20), N=13910 (April 2020), N=10925 (Sep 2020), N=10398 (Jan 2021))

Data on weekly minutes of vigorous activity
(N=14438 (2015-17), N=14427 (2017-2019), N=8981 (2019-20), N=13870 (April 2020), N=11072 (Sep 2020), N=10650 (Jan 2021))

Data on whether 150 mins moderate and 75 mins vigorous activity
(N=13888 (2015-17), N=13504 (2017-2019), N=8071 (2019-20), N=12815 (April 2020), N=10296 (Sep 2020), N=10019 (Jan 2021))

**Age stratification (those aged 16 or over)**(N=13888 (2015-17), N=13504 (2017-2019), N=8071 (2019-20), N=12815 (April 2020), N=10296 (Sep 2020), N=10019 (Jan 2021))

Whether aged 65 or over before March 2020
(N=3502 (2015-17), N=3184 (2017-2019), N=2020 (2019-20), N=2765 (April 2020), N=2656 (Sep 2020), N=2676 (Jan 2021))

16-64
(N=11280 (2015-17), N=10700 (2017-2019), N=6051 (2019-20), N=9770 (April 2020), N7285= (Sep 2020), N=6908 (Jan 2021))

65-73
(N=1867 (2015-17), N=1846 (2017-2019), N=1219 (2019-20), N=1772 (April 2020), N=1712 (Sep 2020), N=1741 (Jan 2021))

74+
(N=741 (2015-17), N=958 (2017-2019), N=801 (2019-20), N=1273 (April 2020), N=1299 (Sep 2020), N=1370 (Jan 2021))

**Deprivation stratification (data on IMD decile)**(N= 3,502 (2015-17), N= 3183 (2017-2019), N=2,020 (2019-20), N=2761 (April 2020), N=2656 (Sep 2020), N=2676 (Jan 2021))

**Health stratification (data on whether health condition)**
(N= 3502 (2015-17), N= 3184 (2017-2019), N= 2020 (2019-20), N= 2765 (April 2020), N= 2656 (Sep 2020), N= 2676 (Jan 2021))

Not deprived
(N=2369 (2015-17), N=2126 (2017-2019), N=1359 (2019-20), N=1882 (April 2020), N=1882 (Sep 2020), N=1883 (Jan 2021))

Deprived
(N=1133 (2015-17), N=1057 (2017-2019), N=661 (2019-20), N=879 (April 2020), N=834 (Sep 2020), N=843 (Jan 2021))

No health condition
(N=1204 (2015-17), N=1089 (2017-2019), N=650 (2019-20), N=943 (April 2020), N=939 (Sep 2020), N=967 (Jan 2021))

Health condition
(N=2298 (2015-17), N=2095 (2017-2019), N=1370 (2019-20), N=1822 (April 2020), N=1717 (Sep 2020), N=1709 (Jan 2021))

Data for at least one wave in the annual study and at least one wave in the COVID-19 study
