# Supplementary material for: The impact of COVID-19 lockdowns on physical activity amongst older adults: evidence from longitudinal data in the UK
Source: BMC Public Health. 2022 Sep 22;22:1802. doi: 10.1186/s12889-022-14156-y (PMC9502942; doi:10.1186/s12889-022-14156-y)

**Additional File 5**

Proportion of older people (aged 65 years and older) meeting physical activity guidelines over time using the alternative proxy for physical activity (red vertical line indicates the introduction of first UK lockdown


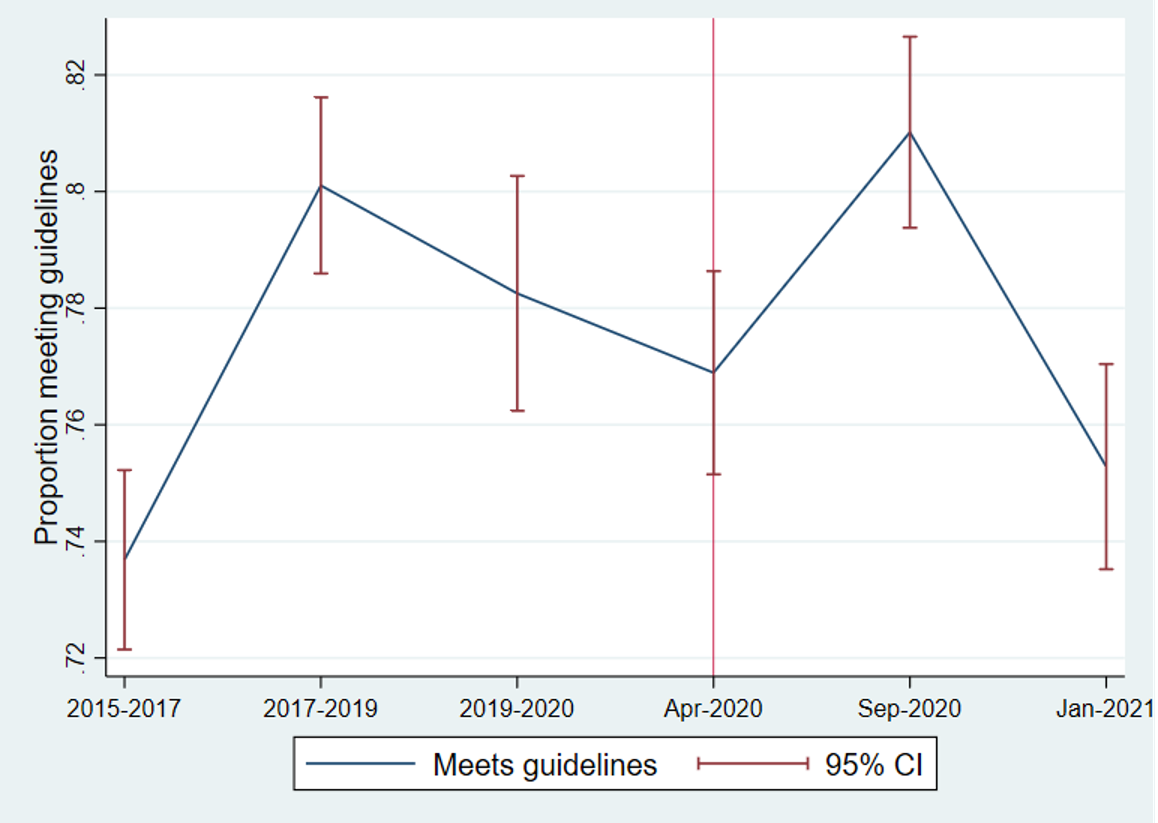

Supplement: Supplementary file 5 — Additional file 5. Proportion of older people (age 65 years and older) meeting physical activity guidelines over time using the alternative proxy for physical activity. [file 12889_2022_14156_MOESM5_ESM.docx]
