# Supplementary material for: The impact of COVID-19 lockdowns on physical activity amongst older adults: evidence from longitudinal data in the UK
Source: BMC Public Health. 2022 Sep 22;22:1802. doi: 10.1186/s12889-022-14156-y (PMC9502942; doi:10.1186/s12889-022-14156-y)

**Additional File 6**

Proportion of older people (aged 65 years and older) meeting physical activity guidelines over time, restricted to the month of data collection


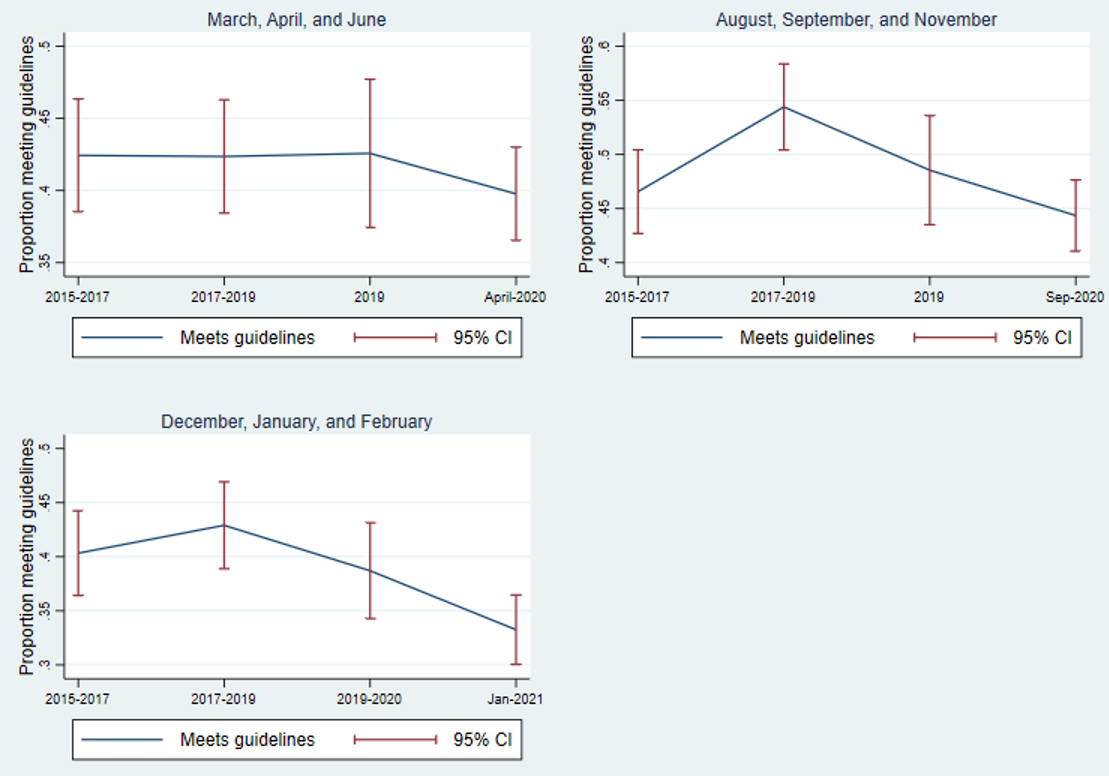

Supplement: Supplementary file 6 — Additional file 6. Proportion of older people (age 65 years and older) meeting physical activity guidelines over time, restricted to the month of data collection. [file 12889_2022_14156_MOESM6_ESM.docx]
